# Analyzing Real-World Infection Risk in Multiple Myeloma Patients Receiving Teclistamab

**DOI:** 10.3390/curroncol33030154

**Published:** 2026-03-08

**Authors:** Paddy Ssentongo, Emma G. Guare, Chen Song, Yoshitaka Inoue, Manpreet Sandhu, Charyguly Annageldiyev, Jeffrey Sivik, Kevin Rakszawski, Seema Naik, Kentaro Minagawa, Shin Mineishi, Catharine I. Paules

**Affiliations:** 1 Division of Infectious Diseases, Department of Medicine, Penn State Health Milton S. Hershey Medical Center, Hershey, PA 17033, USA; csong1@pennstatehealth.psu.edu (C.S.); cpaules@pennstatehealth.psu.edu (C.I.P.); 2 Penn State University College of Medicine, Hershey, PA 17033, USA; egguare@gmail.com; 3 Blood and Marrow Transplant Program, Division of Hematology and Oncology, Penn State Cancer Institute, Hershey, PA 17033, USA; yinoue@pennstatehealth.psu.edu (Y.I.); msandhu@pennstatehealth.psu.edu (M.S.); cannageldiyev@pennstatehealth.psu.edu (C.A.); krakszawski@pennstatehealth.psu.edu (K.R.); snaik@pennstatehealth.psu.edu (S.N.); kminagawa@pennstatehealth.psu.edu (K.M.); smineishi@psu.edu (S.M.); 4 Department of Pharmacy, Penn State Health Milton S. Hershey Medical Center, Hershey, PA 17033, USA; jsivik@pennstatehealth.psu.edu

**Keywords:** teclistamab, bispecific antibody, multiple myeloma, infection, BCMA, real-world data, immunotherapy complications

## Abstract

Background: Teclistamab is an anti-B-cell maturation antigen bispecific antibody used in relapsed, refractory multiple myeloma that induces durable responses but is associated with infectious complications. Real-world data characterizing infection risk remain limited. Methods: We conducted a single-center retrospective cohort study of relapsed/re fractory multiple myeloma patients treated with teclistamab from 1 January 2023 to 20 November 2023. The primary objective was to establish the incidence of infections after initiation of teclistamab. Secondary objectives included infection-related outcomes and identifying potential risk factors for infection. Results: 19 patients received teclistamab with a median age of 72 [IQR: 62–74] years and 73.7% had Karnofsky performance score < 80. A total of 11 (57.9%) patients developed 19 infections, with seven patients having multiple infections. There were five bacteremias, five other bacterial infections, seven respiratory viral infections, and 2 CMV reactivation events. Median time to first infection was 20 days (IQR: 9–87) and median grade of all infections was three (range: 1–5). Of the 19 documented infections, 15 (78.9%) were Grade ≥ 3. A total of 10 patients in the infection group and three in the non-infection group discontinued therapy permanently (*p* = 0.013). Conclusion: In this real-world cohort, infectious complications emerged early and frequently during teclistamab therapy and were a major driver of treatment interruption and permanent discontinuation. The clinical impact of infection extended beyond acute morbidity, often limiting continued access to an otherwise effective therapy in a heavily pretreated population. These findings highlight the need for proactive, individualized infection risk assessment and for standardized, evidence-informed approaches to infection monitoring, prophylaxis, and treatment modification during teclistamab therapy. Larger, multicenter studies will be essential to define strategies that balance infection risk with treatment durability in patients with limited therapeutic alternatives.

## Simple Summary

Teclistamab is an immune-based treatment for relapsed or refractory multiple myeloma that can improve disease control but also increases the risk of infection. We reviewed real-world outcomes of patients treated with teclistamab at our institution to better understand the frequency, timing, and severity of infections. More than half of patients developed at least one infection, many of which were severe and occurred early during therapy. Infections frequently led to hospitalization and were a common reason for permanent discontinuation of teclistamab. These findings highlight the substantial infection burden associated with teclistamab in routine clinical practice and underscore the need for improved strategies to prevent, monitor, and manage infections in patients receiving this therapy.

## 1. Introduction

Bispecific antibodies (BsAbs) are an emerging therapy used to treat B-cell malignancies including relapsed, refractory multiple myeloma (RRMM). Teclistamab is designed to recruit effector T cells to the B-cell maturation antigen (BCMA) thereby enhancing the immune-mediated killing of malignant cells with overall response rates greater than 60% in trial and real-world populations [1,2,3,4]. This activation of the immune system has expected adverse immunologic effects, including hypogammaglobulinemia, pancytopenia, and T cell exhaustion, which when coupled with a heavily pre-treated RRMM population predisposes patients to significant infectious complications [1,5]. We present a descriptive case series highlighting the incidence of infectious complications for patients with RRMM treated with teclistamab at our institution.

## 2. Objective

The objective of this study was to examine the incidence of infections after initiation of teclistamab in a real-world setting. Given the small cohort size, the study was not powered to identify independent predictors of infection, and all comparative findings should be interpreted as exploratory.

## 3. Methods

### 3.1. Data Collection

We performed a single center, systematic, retrospective review of medical charts of patients with RRMM defined as disease progression on three or more lines of prior therapy, who were treated with teclistamab at our institution from 1 January 2023 to 20 November 2023. Electronic medical records were reviewed by authors EGG, CS, MS, and CIP. The primary objective was the incidence of infections after initiation of teclistamab. Secondary objectives included outcomes related to infection and identifying potential risk factors for infection. The study was deemed exempt by the Penn State Cancer Institute institutional review board.

All patients received teclistamab according to step-up subcutaneous dosing of 0.06 mg/kg, 0.3 mg/kg and 1.5 mg/kg given at least 48 h apart in the hospital setting, followed by 1.5 mg/kg weekly outpatient. Dexamethasone 16 mg was given pre-treatment during step-up dosing. Infection prophylaxis with acyclovir or valtrex was given to all patients per institutional standards and pneumocystis jirovecii prophylaxis was given at the primary oncologist’s discretion (see Supplemental Table S1). IVIG was given to patients with a history of sinopulmonary infections, dosed to keep a trough IgG > 400 mg/dL.

Day 0 was defined as the day of the initial teclistamab dose. Infections confirmed by imaging, microbiological (e.g. culture, viral PCR), or histopathological (e.g. biopsy) evidence with correlating clinical symptoms were collected from day 0 to 20 November 2023 or last follow-up. Patients who received empiric antibiotics for presumed cytokine release syndrome (CRS) and immune effector cell-associated neurotoxicity syndrome (ICANS) without the aforementioned criteria were excluded. Those with positive cultures (i.e., urine) but lacking clinical symptoms or documented suspicion for infection were also excluded. Multiple infection events were defined as infections separated by time and site, while co-infections were defined as infections caused by more than one pathogen at the same site and time. Infections were categorized based on organism (bacterial, viral, fungal), site of infection, and graded according to the Common Terminology Criteria for Adverse Events Version 5.0 [6]. Severe outcomes of infection, such as hospitalization, teclistamab discontinuation, and death were reported. Infection events diagnosed outside the Penn State Health system were captured when documentation was available in shared electronic records or communicated through oncology follow-up notes. Outpatient viral infections were identified through documented clinical encounters and confirmatory laboratory testing. No standardized institutional infection surveillance protocol was in place during the study period; testing was performed at provider discretion based on clinical presentation.

Demographic, clinical and laboratory that could be associated with infection were collected. We recorded use of steroids or tocilizumab for CRS/ICANS during step-up and maintenance dosing. Absolute neutrophil count (ANC), absolute lymphocyte count (ALC), and IgG were obtained at Day 0, time of infection or day 20 (median time to first infection) in non-infected patients, and last known follow-up. In patients with IgG kappa MM, IgG levels were adjusted by subtracting the M-spike. Prior lines of therapy and best response teclistamab were defined according to International Myeloma Working Group consensus response criteria [7]. Response to teclistamab was assessed from 4 weeks after initiation through the data cut-off (20 November 2023).

### 3.2. Statistical Analysis

We described the demographic, clinical, laboratory, and post-teclistamab infusion factors among the cohort as a whole and then compared between individuals who developed infection and those who did not. Categorical variables were compared using Fisher’s exact test and reported as frequencies and percentages. Continuous variables were compared using a Wilcoxon rank sum test and reported as median and interquartile range (IQR) or range (minimum and maximum). Further details of each infection event are described in the Supplemental Table S1.

The incidences of infections were calculated using the competing risk model and cumulative incidence estimates. Cumulative curves with 95% CI were generated. Occurrence of infections and death without infections were considered competing events. In all statistical tests, a *p*-value < 0.05 was considered significant. R statistical software (version 4.5.2; R Foundation for Statistical Computing, Vienna, Austria) and EZR (version 1.70; Saitama Medical Center, Jichi Medical University, Saitama, Japan) were used for all analyses. Given the limited sample size, statistical comparisons were exploratory and not adjusted for multiple testing. Emphasis was placed on descriptive patterns and clinical effect direction rather than inferential conclusions.

## 4. Results

19 patients with RRMM received teclistamab therapy (Table 1). The median age of patients was 72 [62–74] years with 10 (52.6%) patients over 70 years and three (15.8%) over 75 years. Nine (47.4%) patients were male and 14 (73.7%) patients were white. Prior to teclistamab treatment, patients had received a median of five previous lines of therapies [4–7]. A total of 14 patients had (73.7%) undergone autologous stem cell transplant (SCT), two (10.5%) had undergone allogeneic SCT, and one (5.3%) had received CAR T therapy. A total of 19 (100%) patients had progressive disease at the time of teclistamab initiation and 14 (73.7%) had Karnofsky performance score < 80. Median follow-up was 196 days (range 39, 315). Patients received infection prophylaxis from the start of teclistamab administration as described in Table 1 and Supplemental Table S1.

**Figure 1:**
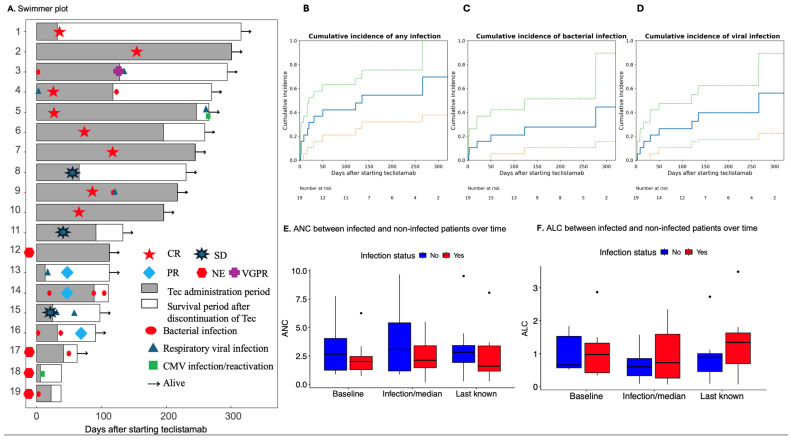
Incidence of infections (**A**–**D**) and ANC/lymphocyte counts between infected and non-infected patients (**E**,**F**) receiving teclistamab. Figure (**A**) Swimmer plot representing each patient in descending order by survival time, denoting infection type during course on teclistamab treatment and best response to treatment. Cumulative incidence curves of any infection (**B**), bacterial infection (**C**), and viral infection (**D**) over time. Comparison of absolute neutrophil count (ANC, (**E**)) and absolute lymphocyte count (ALC, (**F**)) at day 0, or baseline, onset of first infection or day 20 in non-infected patients and last known. Response to teclistamab was assessed 4 weeks after initiation through the data cut-off (20 November 2023). Best response was defined according to International Myeloma Working Group consensus response criteria [7]. CR = complete response, VGPR = very good partial response, PR = partial response, SD = stable disease, and NE = not evaluable.

Eleven (57.9%) patients developed 19 infections with seven patients having more than one infection. There were 5 bacteremias, 5 other bacterial infections (e.g. urinary tract infection, cholangitis, osteomyelitis), seven respiratory viral infections, and two CMV reactivation events without initiation of pre-emptive therapy (Figure 1A, Supplemental Table S1). There were no herpes simplex virus (HSV), varicella zoster virus (VZV), *Pneumocystis* pneumonia (PCP), or fungal infections. One patient had encephalopathy with suspected meningitis/encephalitis, but diagnostic studies could not be obtained, so infection was not confirmed, thus this patient was excluded from the infection group.

The median time to first infection was 20 days (IQR 9, 87) and the cumulative incidence and 95% confidence interval of infection was 36.8% (95%CI 19.6–62.1) at 30 days, 47.4% (95%CI 28.1–71.3) at 100 days, and 68.8% (95%CI 40.7–92.6) at 365 (Figure 1B). The median grade of all infections was 3 (N = 19 total infections; range 1, 5). Of the 19 documented infections, 15 (78.9%) were Grade ≥ 3. Patient characteristics including prior lines of therapy (*p* = 0.90), history of major infection requiring hospitalization prior to teclistamab (*p* = 0.456), ANC at teclistamab start (*p* = 0.484), ALC at teclistamab start (*p* = 0.653), and IgG at teclistamab start (*p* = 0.826) did not vary between the infected and non-infected groups (Figure 1E,F, Table 2). The course of patients on teclistamab including incidence of CRS (*p* = 0.311), use of tocilizumab (*p* = 0.730), or use of IVIG (0.842) did not vary between the infected and non-infected groups, although higher rates of ICANS (54.5% vs. 12.5%) and steroid use for CRS/ICANS (54.5% vs. 25.0%) were observed among infected patients (Table 2). When comparing ANC/lymphocyte counts between infected patients at the time of infection and non-infected patients at 20 days, or the median day to overall infection, ANC (*p* = 0.54), ALC (*p* = 0.54), and IgG (*p* = 1) were similar between groups (Figure 1E,F).

Seven (36.8%) patients developed 10 total bacterial infections, with five cases of bacteremia and five other bacterial infections. Two bacterial infections occurred during step-up dosing. The cumulative incidence of bacterial infections was 15.8% at day 30 and 21.1% at day 100 (Figure 1C). The median time to bacterial infection was 50 days (range 2, 294). One patient developed bacteremia despite being on bacterial prophylaxis during teclistamab administration. At the time of any bacterial infection, median ANC was 1.84 (N = 10, range 0.32, 18.2), median ALC was 0.54 (N = 10, range 0.07, 2.35), and median IgG was 255 (N = 5, range 122, 983). The median grade of bacterial infection was 3 (N = 10, range 2, 5).

Seven (36.8%) patients had nine total viral infections/reactivation events. One viral infection occurred during step-up dosing. The cumulative incidence of viral infections was 15.8% at day 30 and 21.1% at day 100 (Figure 1D). The median time to viral infection was 31 days (range 2, 266). There were seven cases of respiratory viral illness, several coinfections and one patient with multiple respiratory viral illnesses. There were two CMV reactivation events. At the time of any viral infection, median ANC was 3.2 (N = 9, range 0.11, 7.9), median ALC was 1.52 (N = 9, range 0.24, 2.35), and median IgG was 364 (N = 5, range <40, 983). The median grade of viral infection was 1 (N = 9, range 1, 4).

Seven (36.8%) patients were hospitalized for infection and seven infections occurred while patients were in the hospital. A total of 10 patients in the infection group and three in the non-infection group discontinued therapy (*p* = 0.013, Table 3). In the infection group, eight patients discontinued teclistamab due to infection. Three patients in the infection group and 0 in the non-infection group died (*p* = 0.108, Table 3). In the infection group, one patient died from septic shock, one patient died from unknown neurologic causes (ICANS vs. meningitis/encephalitis), and one patient died due to ICANS.

## 5. Discussion

In this descriptive analysis of the first 19 patients at our institution who received teclistamab for RRMM we found a high rate of infectious complications with 57.9% of patients experiencing at least one infection. Risk was cumulative and involved a range of bacterial and viral pathogens. Demographic, clinical, and laboratory features were similar between infected and noninfected patients although there was a trend toward increased ICANS and steroid use for CRS/ICANS among infected patients. Infection resulted in teclistamab discontinuation in 8 patients, hospitalization in 7 patients, extension of hospitalization in 6 patients, and death in 1 patient.

The rapid advancement of novel small molecule targeted therapies and next generation antibody therapies, such as teclistamab, has been accompanied by a dearth of information on potential infectious complications that may arise, and pivotal clinical trials lack detailed reporting of infection events [8]. In the trial that led to the accelerated approval of teclistamab by the Food and Drug Administration (FDA), MajesTEC-1, infections were reported in 76.4% of subjects and grade 3 or 4 infections were reported in 44.8% [2]. Few details were available regarding anatomical location, organism, diagnostic criteria, timing and risk factors for infection prior to widespread availability of the agent on 25 October 2022. In subsequent follow-up of the MajesTEC-1 cohort published in 2023, more nuanced data were provided and confirmed a high infection rate (132/165, 80%; grade 3–4: 91/165, 55.2%) including opportunistic infections (15/165, 9.1%) [9]. No specific risk factors for infection were evaluated, and diagnostic, prophylactic, and management strategies varied by trial site and could not be assessed for efficacy. Thus, subanalyses of trial participants or real-world data remain critical to fill knowledge gaps related to infection after teclistamab. In addition, clinical trial populations differ significantly from real-world populations and may have different infection risk.

In this regard, trial subanalysis and real-world data have confirmed high rates of infection in patients receiving teclistamab, from 31–54% with any infection and 26–55% of infections grade ≥ 3, which is similar to data from our institution [4,10,11]. Our population differs in several discernable ways from not only MajesTEC-1 but also from recently reported real-world analyses [4,10,11]. The median age in our cohort was 72 years (compared to 64 in the trial population and 67–68 in real-world studies) with 3 patients ≥ 75 years [2,4,10,11]. Our patients had poorer functional status with 73.7% of patients at KPS < 80, or ECOG > 1, which would not have met MajesTEC-1 inclusion criteria, and is worse than that reported in other real-world studies (ECOG 2–4 in 33%) [2,10]. Expanding on data from other real-world studies, our median follow-up of 6.4 months is longer than most (3.5–5.5 months) [4,10,11], which is valuable because MajesTEC-1 reported an increased cumulative risk of infection with longer therapy [9,12]. While it is reassuring that overall infection rates in our cohort are similar to those reported in MaJesTEC-1 and in real-world analyses, infections were more severe with a median grade of 3, and 11 (57.9%) infections were grade ≥ 3. Additionally, 7/19 patients (36.8%) had recurrent infections. One real-world study similarly reported high rates of multiple infections with 78 total infections in 44 patients [4], although multiple infection events are reported infrequently and inconsistently. While it remains difficult to assess the baseline health of populations by means of functional status, age, and comorbidities, it is possible that our population was sicker overall, resulting in multiple and more severe infections. Additionally, high rates of CRS (63.2%) and ICANS (36.8%) were seen among our cohort, with 36.8% necessitating steroids, which is higher than rates of steroid use in other real-world studies (17–33%) [4,10]. There was also a trend towards increased ICANS (*p* = 0.061) and steroid use (0.63) among infected patients, which has been noted in other real-world studies [13]. These findings necessitate further exploration as teclistamab is utilized more broadly. Beyond serving as adverse events, CRS and ICANS may also interact biologically with infection risk. Infections and CRS/ICANS may be bidirectionally related during teclistamab therapy. Infections can serve as inflammatory triggers that augment cytokine release and potentially exacerbate CRS or neurotoxicity. Conversely, immunosuppressive treatments used to manage CRS/ICANS, particularly corticosteroids, may increase susceptibility to subsequent infections. Although causal relationships could not be established in this small retrospective cohort, we observed higher rates of ICANS and steroid use among patients who developed infections, supporting a potential interaction between immune toxicity, its management, and infection risk.

Our findings suggest that hypogammaglobulinemia may play a more central role in infection risk during teclistamab therapy than neutropenia. In this cohort, we did not observe an association between absolute neutrophil count and the incidence of bacterial infection, whereas IgG levels were frequently markedly reduced at the time of infection. This pattern is biologically plausible given teclistamab’s on-target depletion of normal plasma cells and impairment of humoral immunity. These data support emerging evidence that antibody deficiency, rather than myelosuppression alone, is a key driver of infection risk in patients receiving BCMA-directed bispecific antibodies and underscore the potential importance of early and consistent immunoglobulin replacement strategies [14].

Infection was grounds for permanently discontinuing teclistamab in 8/19 patients in our study. Real-world studies seldom discuss reasons for teclistamab discontinuation, with anywhere between 5–13% permanently discontinuing teclistamab for infection [10,13]. In our dataset, the most common infection-related reasons for discontinuation included multiple infections, grade 3+ infections, and long-term impairments from infection. In addition, 1 patient died from infection and two developed significant disease progression after teclistamab was stopped for infection. In a patient population with few treatment options, understanding when to interrupt, lengthen dosing intervals or permanently discontinue salvage therapy is critically important. Additionally, as infection was the most common cause of treatment cessation and death in our population, standardized infection prevention and management strategies are critically needed. In this small cohort, we were unable to detect a measurable association between antimicrobial prophylaxis or IVIG use and infection risk, however, these findings are inconclusive given limited power and practice variability, and require validation in larger, multicenter studies. Real-world data from other studies have been mixed and retrospective in nature [4,10,12,15]. While a few expert groups have outlined recommendations regarding IVIG supplementation, antimicrobial prophylaxis, neutropenia management, and vaccination for patients receiving teclistamab, guidance is based largely on expert opinion and very limited clinical data [14,16]. Dedicated, rigorous studies analyzing the infection risk of teclistamab in real-world recipients are thus warranted as well as comparison of infection rates, type, and timing with other salvage regimens for multiple myeloma.

Our study has several important limitations, primarily including its retrospective nature, heterogeneity of clinical practice between providers, and small sample size. Within our institution, 5 different physicians prescribe teclistamab with differing approaches to clinical practice for both multiple myeloma and infection. Variation included differences in pneumocystis prophylaxis selection and duration, IVIG initiation thresholds (history-based vs. IgG threshold–based), frequency of outpatient viral testing, and timing of laboratory monitoring. These practice differences may have influenced both infection detection and management decisions, including hospitalization and treatment interruption. In addition, several patients involved in this study receive teclistamab at community oncology practices after initial step-up dosing. Therefore, an important limitation includes variation in physician practice patterns and inconsistent follow-up times that may have impacted the calculated rate of infection and resulted in differences in teclistamab discontinuation between providers. Data were insufficient to comprehensively analyze risk factors for infection or to provide data regarding mitigation and treatment strategies. While data such as ours are valuable and can be aggregated with experience at other centers, the limitations above highlight the need for more robust data collection systems to address gaps in knowledge [8]. Groundbreaking cancer therapies such as teclistamab are being granted accelerated approval, yet the acceptable and expected rate of infection along with effective prevention strategies must be defined concurrently and go beyond the documentation of infection as graded adverse events. This may require leveraging large multicenter registries to evaluate infection risk in more nuanced ways, standardizing infection definitions across databases, and eventually designing clinical trials to more effectively assess this outcome. In addition, designing post-marketing studies of novel oncology therapies such as teclistamab to assess infection prevention and management strategies in real-world populations is crucial.

## 6. Conclusions

In this real-world cohort, infectious complications emerged early and frequently during teclistamab therapy and were a major driver of treatment interruption and permanent discontinuation. The clinical impact of infection extended beyond acute morbidity, often limiting continued access to an otherwise effective therapy in a heavily pretreated population. These findings highlight the need for proactive, individualized infection risk assessment and for standardized, evidence-informed approaches to infection monitoring, prophylaxis, and treatment modification during teclistamab therapy. Larger, multicenter studies will be essential to define strategies that balance infection risk with treatment durability in patients with limited therapeutic alternatives.

## Figures and Tables

**Table 1:** All patient characteristics.

Characteristics		N = 19 ^a^
Age at teclistamab start	median [IQR], years	72 [62, 74]
	≥70 years	10 (52.6)
	≥75 years	3 (15.8)
Sex	Female	10 (52.6)
	Male	9 (47.4)
Race/ethnicity	White	14 (73.7)
	Hispanic	4 (21.1)
	Black persons	1 (5.3)
Karnofsky performance status	≥80	5 (26.3)
	<80	14 (73.7)
Prior lines of therapy	Median [IQR]	5 [4, 7]
Prior transplant	Autologous SCT	14 (73.7)
	Allogeneic SCT	2 (10.5)
	CAR-T	1 (5.3)
Major infection ^b^ in 12mo before teclistamab		8 (42.1)
ANC < 500 in 12mos before teclistamab		6 (31.6)
ANC at teclistamab start	median [IQR],/μL	2050 [1260, 3320]
ALC at teclistamab start	median [IQR],/μL	720 [520, 1405]
	<1000/μL	11 (57.9)
IgG at teclistamab start ^c^	median [IQR] mg/dL	427.5 [326, 667.8]
	<400 mg/dL	8 (42.1)
Use of IVIG		9 (47.4)
Use of bacterial ppx		3 (15.8)
Use of viral ppx		19 (100)
Use of PCP ppx		8 (42.1)
Use of fungal ppx		2 (10.5)
CRS (all grades)		12 (63.2)
≥G3 CRS		1 (5.3)
ICANS (all grades)		7 (36.8)
≥G3 ICANS		2 (10.5)
Use of steroid for CRS/ICANS		7 (36.8)
Use of tocilizumab for CRS/ICANS		8 (42.1)
Best response to teclistamab	CR	8 (42)
	VGPR	1 (5)
	PR	3 (15.8)
	SD	3 (15.8)
	NE	4 (21.0)

Abbreviations: SCT, stem cell transplant; CAR-T, chimeric antigen receptor-T cell; ANC, absolute neutrophil count; ALC, absolute lymphocyte count; IgG, immunoglobulin G; IVIG, intravenous immunoglobulin; ppx, prophylaxis; PCP, *Pneumocystis* pneumonia; CRS, cytokine release syndrome; ICANS, immune effector cell-associated neurotoxicity syndrome. Response to teclistamab was assessed from 4 weeks after initiation through the data cut-off (20 November 2023). Best response was defined according to International Myeloma Working Group consensus response criteria [7]. CR = complete response, VGPR = very good partial response, PR = partial response, SD = stable disease, and NE = not evaluable; ^a^ Unless otherwise indicated, data are expressed as No. (%); ^b^ Major infection refers to infection confirmed by imaging, microbiological, or histopathological evidence, requiring hospitalization; ^c^ In 7 patients with IgG kappa MM, IgG levels were adjusted by subtracting the M-spike.

**Table 2:** Characteristics between infected and non-infected patients.

Characteristics		Any Infection After Teclistamab ^a^		***p*** Value
		No	Yes	
		8	11	
Age at teclistamab start	median [range], years	70 [57, 82]	72 [52, 80]	0.562
	≥70 years	4 (50.0)	6 (54.5)	0.844
	≥75 years	2 (25.0)	1 (9.1)	0.348
Sex	Female	3 (37.5)	7 (63.6)	0.260
	Male	5 (62.5)	4 (36.4)	
Race/ethnicity	White	7 (87.5)	7 (63.6)	0.098
	Hispanic	0 (0)	4 (36.4)	
	Black persons	1 (12.5)	0 (0)	
Karnofsky performance status	≥80	2 (25.0)	3 (27.3)	0.913
	<80	6 (75.0)	8 (72.7)	
Prior line of therapy	median (range)	5 [2, 9]	5 [2, 8]	0.90
Prior transplant	Autologous SCT	7 (87.5)	7 (63.6)	0.331
	Allogeneic SCT	1 (12.5)	1 (9.1)	
	CAR-T	1 (12.5)	0 (0)	
Infection in 12 months before teclistamab		4 (50.0)	4 (36.4)	0.456
ANC < 500 in 12mos before teclistamab ^c^		2	4	0.598
ANC at teclistamab start ^c^	median [range],/μL	2670 [890, 7760]	2010 [730, 6260]	0.484
ALC at teclistamab start ^c^	median [range],/μL	660 [520, 1840]	980 [340, 2870]	0.653
	<1000/μL	5 (62.5)	6 (54.5)	0.730
IgG count before teclistamab ^c^	median [range],/mg	418.5 [227, 1111]	427.5 [157, 1697]	0.826
	<400/mg	4 (50.0)	4 (10.0)	
	≥400/mg	4 (50.0)	6 (80.0)	
	Unknown	0 (0.0)	1 (10.0)	
Use of IVIG		4 (50.0)	5 (45.5)	0.842
Use of bacterial ppx		2 (25.0)	1 (9.1)	0.347
Use of viral ppx		8 (100)	11 (100)	1
Use of PCP ppx		3 (37.5)	5 (45.4)	0.730
Use of fungal ppx		1 (12.5)	1 (9.1)	0.810
CRS (all grades)		4 (50.0)	8 (72.7)	0.311
ICANS (all grades)		1 (12.5)	6 (54.5)	0.061
Use of steroid for CRS/ICANS		2 (25.0)	5 (45.5)	0.63
Use of tocilizumab for CRS/ICANS		3 (37.5)	5 (45.5)	0.730

Abbreviations: SCT, stem cell transplant; CAR-T, chimeric antigen receptor-T cell; ANC, absolute neutrophil count; ALC, absolute lymphocyte count; IgG, immunoglobulin G; IVIG, intravenous immunoglobulin; ppx, prophylaxis; PCP, *Pneumocystis* pneumonia; CRS, cytokine release syndrome; ICANS, immune effector cell-associated neurotoxicity syndrome. ^a^ Unless otherwise indicated, data are expressed as No. (%); ^c^ ANC and ALC expressed in K/uL, IgG: Immunoglobulin G expressed in mg/dL.

**Table 3:** Patient outcomes on teclistamab between infected and non-infected patients.

Number of Cases ^a^	Non-Infected	Infected	*p* Value
	8	11	
Discontinued teclistamab	3 (37.5)	10 (90.9) ^b^	0.013
Deaths	0 (0)	3 (27.3) ^c^	0.108

^a^ Unless otherwise indicated, data are expressed as No. (%); ^b^ 8 patients discontinued teclistamab due to infection; ^c^ 1 death was attributed to infection.

## Data Availability

The data that support the findings of this study are not publicly available due to patient privacy and institutional restrictions but are available from the corresponding author on reasonable request and with approval from the Penn State Cancer Institute Institutional Review Board.

## Supplementary Materials

The following supporting information can be downloaded at: https://www.mdpi.com/article/10.3390/curroncol33030154/s1, Table S1: Details regarding infection history, type of infection during teclistamab, and teclistamab discontinuation amongst infected patients.

## Abbreviations

The following abbreviations are used in this manuscript:

ABSSSI	Acute bacterial skin and skin structure infection
ALC	Absolute lymphocyte count
ANC	Absolute neutrophil count
BCMA	B-cell maturation antigen
CAR-T	Chimeric antigen receptor T-cell
CMV	Cytomegalovirus
CR	Complete response
CRS	Cytokine release syndrome
ICANS	Immune effector cell–associated neurotoxicity syndrome
IgG	Immunoglobulin G
IQR	Interquartile range
IVIG	Intravenous immunoglobulin
KPS	Karnofsky Performance Status
MM	Multiple myeloma
PCP	*Pneumocystis jirovecii* pneumonia
RRMM	Relapsed/refractory multiple myeloma
SCT	Stem cell transplant
VGPR	Very good partial response
