# Supplementary material for: Analyzing Real-World Infection Risk in Multiple Myeloma Patients Receiving Teclistamab
Source: Curr Oncol. 2026 Mar 8;33(3):154. doi: 10.3390/curroncol33030154 (PMC13025476; doi:10.3390/curroncol33030154)
Supplement: Supplementary file 1 [file curroncol-33-00154-s001.zip › curroncol-4118538-supplementary.pdf]

Analyzing Real-World Infection Risk in Multiple Myeloma Patients Receiving Teclistamab

<u>Supplementary Material</u>

Supplemental Table S1. Details regarding infection history, type of infection during teclistamab, and teclistamab discontinuation amongst infected patients

Case	Infection in 12 months prior to teclistamab	Antimicrobial prophylaxis	Infection during teclistamab	Grade of infection	Day of infection	Immune markers at time of infection	Hospitalized for infection / death	Teclistamab discontinued due to infection	Best response
3	no	valacyclovir	UTI	Grade 3	2	ANC 1.73, ALC 0.44	Dev in hospital	yes	VGPR
			COVID-19 and rhinovirus/enterovirus coinfection	Grade 3	135	ANC 0.11, ALC 2.06, IgG 364	hospitalized		
4	no	acyclovir	rhinovirus/enterovirus infection	Grade 1	3	ANC 5.06, ALC 0.24	Dev in hospital	yes	CR
			Haemophilus haemolyticus bacteremia	Grade 3	123	ANC 18.2, ALC 0.62, IgG 122	hospitalized		
5	no	Acyclovir, TMP/SMX	rhinovirus/enterovirus infection	Grade 4	266	ANC 0.17, ALC 1.89, IgG <40	hospitalized to ICU	no	CR
			CMV reactivation	Grade 1	275	ANC 8.64, ALC 1.52	Dev in hospital		
9	COVID-19 treated with paxlovid	acyclovir	Rhinovirus/enterovirus infection	Grade 1	118	ANC 3.20, ALC 2.35, IgG 983	no	no	CR
			odontogenic (bacterial) jaw infection	Grade 2	118	ANC 3.20, ALC 2.35, IgG 983	no		
13	no	valacyclovir	adenovirus and COVID-19 coinfection	Grade 3	17	ANC 2.12, ALC 0.73	hospitalized	yes	PR

14	COVID-19 treated with paxlovid	penicillin V, levofloxacin, doxycycline; valacyclovir; atovaquone; posaconazole	*Pseudomonas aeruginosa* bacteremia	Grade 3	20	ANC 2.13, ALC 0.14, IgG 525	hospitalized	yes	PR
			Staphylococcus epidermidis bacteremia	Grade 4	88	ANC 0.63, ALC 0.46, IgG 905	Hospitalized to ICU		
			Pseudomonas aeruginosa bacteremia	Grade 5	104	ANC 0.32, ALC 0.65	Dev in hospital, death		
15	no	acyclovir	adenovirus infection	Grade 2	31	ANC 5.5, ALC 0.28	ED	yes	SD
			rhinovirus/enterovirus infection	Grade 1	74	ANC 7.9, ALC 0.37, IgG 82	no		
16	osteomyelitis and underwent toe amputation	Acyclovir, TMP/SMX	coagulase negative S*taphylococcus* bacteremia	Grade 3	2	ANC 3.58, ALC 0.07	Dev in hospital	yes	PR
			Osteomyelitis	Grade 3	37	ANC 1.95, ALC 0.25	hospitalized		
17	no	Acyclovir, TMP/SMX	cholangitis	Grade 4	50	ANC 0.87, ALC 1.3, IgG 191	hospitalized	yes	NE
18	coinfection with COVID-19 and rhinovirus/enterovirus requiring hospitalization	Famciclovir, atovaquone	CMV reactivation	Grade 1	9	ANC 1.87, ALC 1.58, IgG 885	Dev in hospital	yes	NE
19	no	acyclovir	UTI	Grade 2	3	ANC 1.18, ALC 1.6	Dev in hospital	no	NE

Abbreviations: ANC: absolute neutrophil count; ALC: absolute lymphocyte count; ANC and ALC expressed in K/uL, IgG: Immunoglobulin G expressed in mg/dL; UTI: urinary tract infection; dev: developed; TMP/SMX: trimethoprim sulfamethoxazole; ICU: intensive care unit; ED: emergency department

Infections were confirmed by imaging, microbiological, or histopathological evidence with clinical correlation, and were graded according to the Common Terminology Criteria for Adverse Events Version 5.0[6]

Response to teclistamab was assessed from 4 weeks after initiation through the data cut-off (11/20/2023). Best response was defined according to International Myeloma Working Group consensus response criteria[7]. CR=complete response, VGPR= very good partial response, PR= partial response, SD=stable disease, and NE =not evaluabl
